# A new terrestrial talitrid genus, *Kachinorchestia*, with a new species from Myanmar (Crustacea, Amphipoda, Arcitalitridae)

**DOI:** 10.3897/BDJ.13.e162403

**Published:** 2025-10-13

**Authors:** Yan Tong, Zeyu Liu, Hongguang Liu, Zhonge Hou

**Affiliations:** 1 State Key Laboratory of Animal Biodiversity Conservation and Integrated Pest Management, Institute of Zoology, Chinese Academy of Sciences, Beijing, China State Key Laboratory of Animal Biodiversity Conservation and Integrated Pest Management, Institute of Zoology, Chinese Academy of Sciences Beijing China; 2 University of Chinese Academy of Sciences, Beijing, China University of Chinese Academy of Sciences Beijing China

**Keywords:** landhopper, morphology, DNA barcodes, new genus, taxonomy

## Abstract

**Background:**

The Arcitalitridae (Amphipoda, Senticaudata, Talitrida, Talitroidea) is a diverse family containing 15 genera, of which *Myanmarorchestia* Hou, 2017 and *Solitroides* Suzuki, Nakano, Nguyen, Nguyen, Morino & Tomikawa, 2017 were reported in Southeast Asia. During research of terrestrial amphipods in Myanmar, we found character traits of some specimens did not match any existing genus of the family Arcitalitridae. These specimens should belong to a new genus.

**New information:**

This new genus *Kachinorchestia* Hou, gen. nov. with one new species *Kachinorchestia putao* Hou, sp. nov. is described from terrestrial habitats in Myanmar. This new genus is characterised by mandible left lacinia mobilis with four teeth, gnathopod II propodus of male enlarged, oval and subchelate, with hook posteriorly, simplidactylate pereopods, complex and lobed gills and telson uncleft. Photos, molecular and morphological descriptions of new genus are provided.

## Introduction

Myers & Lowry (2020) recently published a revision of the Talitroidea, which erected two epifamilies, the Talitroidae and Protorchestoidae. The Protorchestoidae includes three families, Arcitalitridae, Protorchestiidae and Uhlorchestiidae (Myers and Lowry 2020). The Arcitalitridae is characterised by maxilliped palp article 2 without distomedial lobe, article 4 small, distinct; pereopods III–VII simplidactylate, uropod I outer ramus without marginal robust setae and uropod 3 ramus shorter than peduncle. Currently there are 15 genera in the family Arcitalitridae, of which two terrestrial genera have been recorded in the Southeast Asia. The genera *Solitroides* Suzuki, Nakano, Nguyen, Nguyen, Morino & Tomikawa, 2017 were recorded in Vietnam (Suzuki et al. 2017), while *Myanmarorchestia* Hou, 2017 were found in Myanmar. Here, we assign a new genus *Kachinorchestia* to Arcitalitridae, due to its maxilliped palp article 2 without distomedial lobe, article 4 small, distinct; pereopods III–VII simplidactylate and uropod III ramus shorter than peduncle. The detailed relationships amongst these morphological groups need to be assessed with molecular evidence.

**Figure 1.**
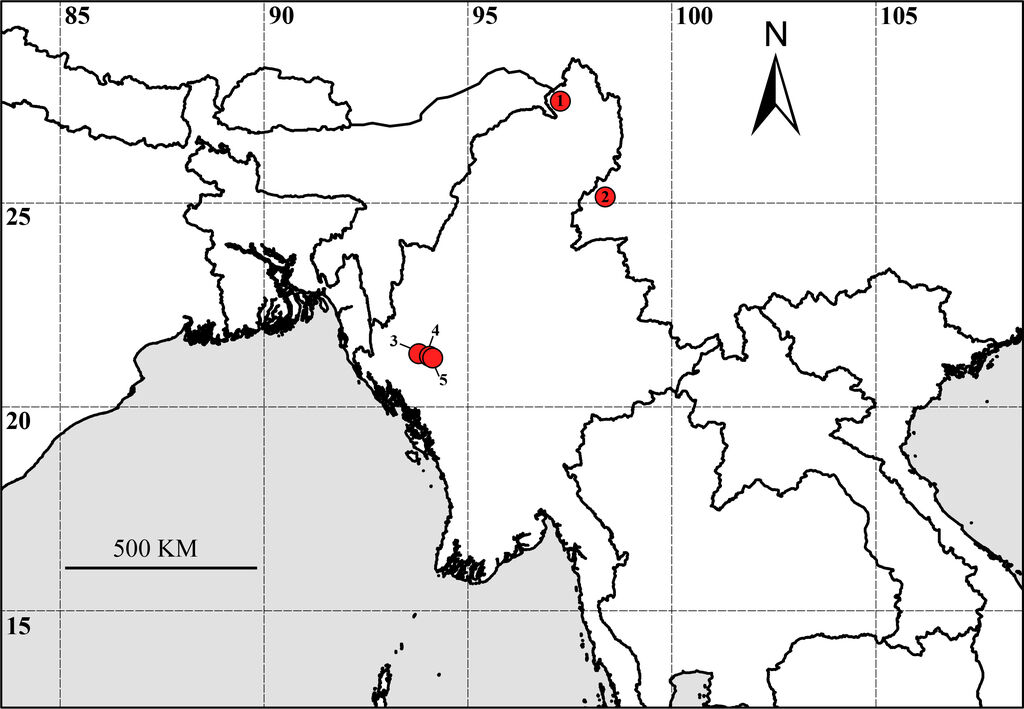
Type localities of *Kachinorchestia* and *Myanmarorchestia* species from Myanmar and China. 1. *K. putao* sp. nov. 2. *M. nunomurai* Nakano & Morino, 2018; 3. *M. victoria* Hou, 2017; 4. *M. peterjaegeri* Hou, 2017; 5. *M. seabri* Hou, 2017.

The landhopper genus *Myanmarorchestia* Hou in Hou and Zhao (2017) is most recognisable by coxal gills convoluted, with or without marginal filamentous projections and has a well-defined distribution. The new genus *Kachinorchestia* is most similar to the genus *Myanmarorchestia* in coxal gills convoluted. The convoluted gills might be some of the adaptations to terrestrial habitat (Liu et al. 2025). It is mainly recorded in Southeast Asia (Liu et al. 2023). The genus *Myanmarorchestia* currently includes four species, of which three species have been recorded in high altitude forests of Mt. Victoria, Myanmar and show some vertical distribution patterns. For example, *M. victoria* Hou in Zheng and Hou (2017) occurs above 2600 m above sea level, *M. peterjaegeri* Hou in Hou and Zhao (2017) occurs above 2000 m, while *M. seabri* Hou in Hou and Zhao (2017) inhabits understorey leaf litter around 1500 m. The last species *M. nunomurai* (Nakano et al. 2018) was recorded in the mountainous habitat (elevation 1900 m) of Yunnan, China, near the border of Myanmar (Fig. 1).

During a field survey of terrestrial amphipods in Myanmar, landhoppers were collected from mountain forest habitats. In this paper, these landhoppers are described as a new genus *Kachinorchestia* Hou, gen. nov. and a new species, *Kachinorchestia putao* Hou, sp. nov. The barcode sequence of the new species is presented and the genetic distances between the new species and known species are calculated to affirm the species delimitation.

## Materials and methods

**Morphological observation**

The specimens were collected by sweeping rotten wood with a fine-meshed hand net. Samples were preserved in 95% ethanol in the field and deposited under -20°C refrigeration for long-term preservation. The amphipod’s body length was recorded by holding the specimen straight and measuring the distance along the dorsal side of the body from the base of the first antenna to the base of the telson (Suzuki et al. 2017, Wang et al. 2024). Photographs were taken using an Olympus C7070 wide zoom digital camera. Helicon Focus version 6.0 software was used for digital stacking, while the final images were edited and polished using Adobe’s Photoshop cc 2020 and Illustrator 2021 software. Appendages were drawn using a Leica DM2500 compound microscope, equipped with a drawing tube. Terminology and taxonomic descriptions follow literature (Hou and Zhao 2017, Zheng and Hou 2017). The terms “setae” and “spines” are used to distinguish between thin or fine and more robust setal structures. All types and other materials are lodged in the Institute of Zoology, Chinese Academy of Sciences (IZCAS), Beijing.

**DNA sequencing and phylogenetic analyses**

Genomic DNA was extracted from appendages of the specimens using a TIANamp Genomic DNA Kit (TIANGEN). The fragments of the 28S and COI were amplified and sequenced following published protocols (Hou et al. 2022). The new sequences and reference sequences downloaded from GenBank were aligned using MEGA v.7.0.26 (Kumar et al. 2016). The GenBank accession numbers and pairwise distances are presented in Table 1 and Table 2. In total, 24 specimens belonging to 17 talitrid species, were used in molecular phylogenetic analyses. The *Hyalella tiwanaku* Gonzalez & Watling, 2003 and *Hyalella franciscae* Gonzalez & Watling, 2003 were selected as outgroup taxa. Phylogenetic relationships were inferred using Maximum Likelihood (ML) and Bayesian Inference (BI) on concatenated sequences. ML phylogenies were conducted using RAxML v.8.2.12 (Stamatakis 2014) with 1000 rapid bootstrap replicates followed by a thorough tree search. Bayesian analyses were carried out using MrBayes v.3.2.7 (Ronquist et al. 2012), implementing two independent runs of five million generations.

## Taxon treatments

### Kachinorchestia

Hou gen. nov.

5F112ECC-75AD-591D-8729-21A93BF80ED6

157F7226-7246-4D6A-AD0E-29A8FB615215

Kachinorchestia putao Hou. Type species.

#### Diagnosis

Body size medium. Eyes rounded. Antenna I reaching mid-point of peduncular article V of antenna II, flagellum a little shorter than peduncle. Antenna II flagellum a little longer than peduncle. Mandible left lacinia mobilis with four teeth; right lacinia mobilis bifurcate. Maxilla I outer plate with one small articulate palp present. Maxilliped palp article 4 reduced, small, but distinct.

Gnathopod I sexually dimorphic; merus and carpus of male protuberant medioposteriorly, propodus distally expanded and subchelate, merus and carpus of female without protuberance, propodus simple. Gnathopod II sexually dimorphic; propodus of male enlarged, oval and subchelate, with hook posteriorly and spines, propodus of female expanded, with protuberance posteriorly. Pereopods simplidactylate, with two spines at hinge of unguis. Coxal gills present on gnathopod II and pereopods III–VI; each gill lobed and convoluted, without ridged margins or filamentous projections. Oostegites slender, present on gnathopod II and pereopods III–V.

Epimeral plates acuminate posterodistally, distal margins without armature. Pleopods well-developed, peduncles with plumose setae on exterior margins. Uropod I peduncle with distolateral spine, outer ramus marginally bare. Uropod III ramus narrower and shorter than peduncle. Telson uncleft, with one terminal spine each side.

The general morphology is stable across all examined male specimens, with variations only in quantitative characters like the number of spines.

#### Etymology

The generic name is derived from “Kachin” the State of type locality, in combination with the Orchestia stem.

### Kachinorchestia putao

Hou sp. nov.

7FD677BB-A901-5213-AF6F-6BF566645AB7

E43793A0-B3CB-4AE5-A1C3-73C334D3C275

#### Materials

- **Type status:** Holotype. **Occurrence:** recordedBy: Wu Jiang-lang, Chen Zhi-gang; individualCount: 1; sex: male; lifeStage: adult; occurrenceID: A3361FE6-5E9B-5DFB-BCE6-5593D45D01A2; **Taxon:** scientificName: *Kachinorchestia putao* Hou, sp. nov.; order: Amphipoda; family: Arcitalitridae; genus: Kachinorchestia; **Location:** country: Myanmar; stateProvince: Kachin State; county: Putao; locality: Hponkanrazi Wildlife Sanctuary; verbatimElevation: 1447 m; verbatimCoordinates: 27°35.526'N 97°01.303'E; decimalLatitude: 27.59; decimalLongitude: 97.02; **Record Level:** institutionCode: IZCAS-I-A2096-1
- **Type status:** Paratype. **Occurrence:** recordedBy: Wu Jiang-lang, Chen Zhi-gang; individualCount: 1; sex: female; lifeStage: adult; occurrenceID: 307A74BD-DC01-51D9-9612-461615F2D222; **Taxon:** scientificName: *Kachinorchestia putao* Hou, sp. nov.; order: Amphipoda; family: Arcitalitridae; genus: Kachinorchestia; **Location:** country: Myanmar; stateProvince: Kachin State; county: Putao; locality: Hponkanrazi Wildlife Sanctuary; verbatimElevation: 1447 m; verbatimCoordinates: 27°35.526'N 97°01.303'E; decimalLatitude: 27.59; decimalLongitude: 97.02; **Record Level:** institutionCode: IZCAS-I-A2096-2
- **Type status:** Paratype. **Occurrence:** recordedBy: Wu Jiang-lang, Chen Zhi-gang; individualCount: 1; sex: male; lifeStage: adult; occurrenceID: DE312ECB-DFA1-5419-80E9-AB2451719CC4; **Taxon:** scientificName: *Kachinorchestia putao* Hou, sp. nov.; order: Amphipoda; family: Arcitalitridae; genus: Kachinorchestia; **Location:** country: Myanmar; stateProvince: Kachin State; county: Putao; locality: Hponkanrazi Wildlife Sanctuary; verbatimElevation: 1447 m; verbatimCoordinates: 27°35.526'N 97°01.303'E; decimalLatitude: 27.59; decimalLongitude: 97.02; **Record Level:** institutionCode: IZCAS-I-A2096-3

#### Description

**Figure 2.**
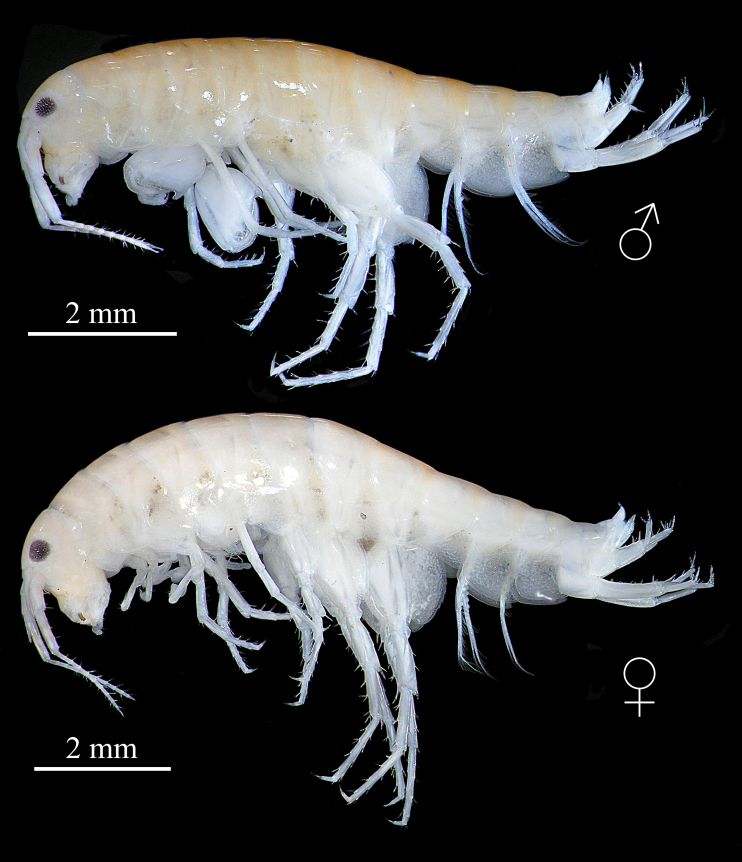
*Kachinorchestia putao* sp. nov. holotype male, 8.0 mm and paratype female, 9.0 mm.

Based on holotype male (IZCAS-I-A2096-1), 8.0 mm (Fig. 2).

**Figure 3.**
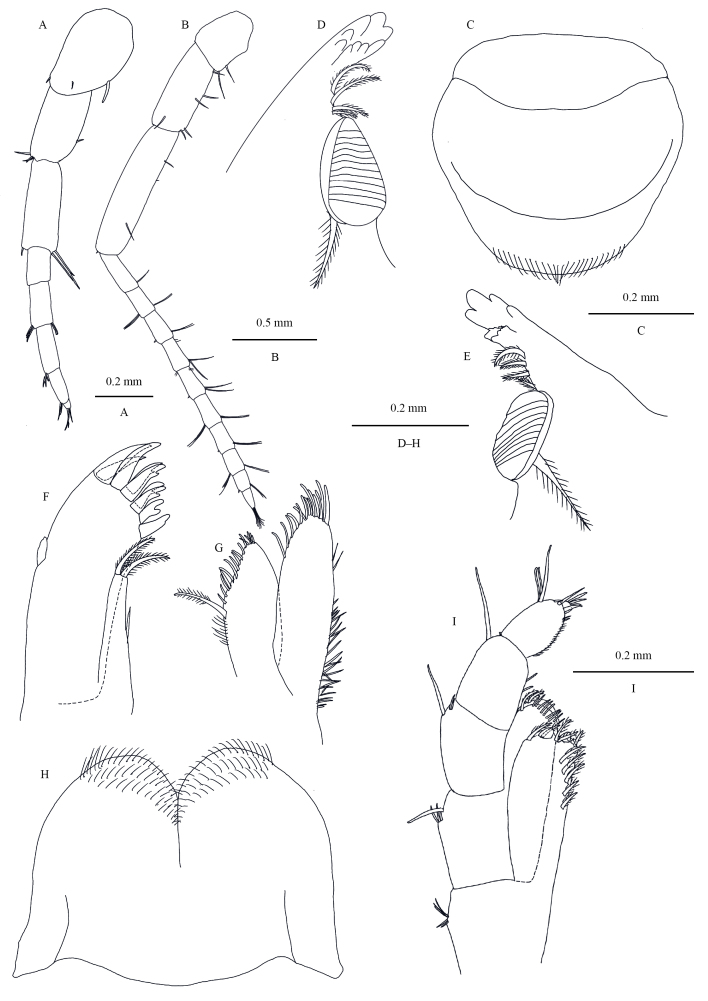
*Kachinorchestia putao* sp. nov. holotype male **A** antenna I; **B** antenna II; **C** upper lip; **D** left mandible; **E** right mandible; **F** maxilla I; **G** maxilla II; **H** lower lip; **I** maxilliped.

**Head.** Eyes rounded (Fig. 2). Antenna I (Fig. 3A): reaching mid-point of peduncular article V of antenna II, flagellum a little shorter than peduncle. peduncle articles 1–3 in length ratio 1.0: 1.0: 1.1, with distal spines; flagellum with five articles, last article tiny, each article with short distal setae. Antenna II (Fig. 3B): peduncle articles 3–5 in length ratio 1.0: 2.3: 3.2; flagellum with 10 articles. Upper lip (Fig. 3C): ventral margin rounded, bearing minute setae. Mandible (Fig. 3D and E): left mandible incisor with five teeth; lacinia mobilis with four teeth; spine row with four plumose setae; molar well developed and with a plumose seta; right mandible incisor with five teeth, lacinia mobilis bifurcate. Lower lip (Fig. 3H): inner lobes indistinct, outer lobes covered with fine setae. Maxilla I (Fig. 3F): inner plate thin, with two terminal plumose setae; outer plate with nine terminal serrate setae; one small articulate palp present. Maxilla II (Fig. 3G): inner plate narrower and shorter than outer plate, with one plumose seta and numerous simple setae on medial margin; outer plate with two rows of apical setae. Maxilliped (Fig. 3I): inner plate with three stout spines distally; outer plate with apical setae; palp article 4 reduced, small, but distinct.

**Figure 4.**
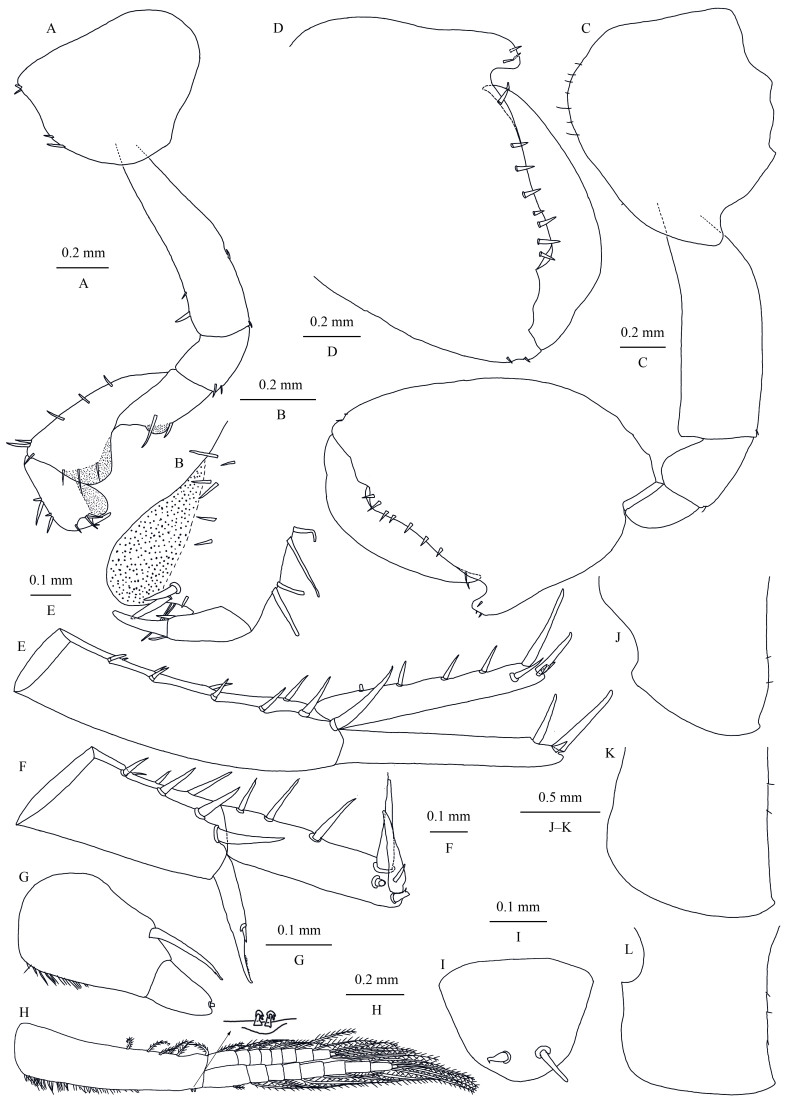
*Kachinorchestia putao* sp. nov. holotype male **A** gnathopod I; **B** propodus of gnathopod I; **C** gnathopod II; **D** propodus of gnathopod II; **E** uropod I; **F** uropod II; **G** uropod III; **H** pleopod I; **I** telson; **J** epimeral plate I; **K** epimeral plate II; **L** epimeral plate III.

**Pereon.** Gnathopod I (Fig. 4A and B): sexually dimorphic; coxal plate ventral margin rounded, with four small spines; basis anterior and posterior margins with short setae; merus, carpus and propodus in length ratio 1.0: 1.9: 1.1; merus protuberant medioposteriorly, bearing two spines on the base of the protuberance; carpus anterior and posterior margins with setae; propodus distally expanded, with a row of spines on both faces, palm margin well developed exceeding dactylus length, with two spines medially; dactylus with two setae at hinge of unguis. Gnathopod II (Fig. 4C and D): sexually dimorphic; coxal plate wider than that of gnathopod I, with a short posterior cusp, ventral margin spinose; basis stout, posterior margin with a minute spine; propodus enlarged and oval, 1.3 times as long as wide, with hook posteriorly and spines; palmar margin smoothly convex, fringed with spines; dactylus as long as propodus palm.

**Figure 5.**
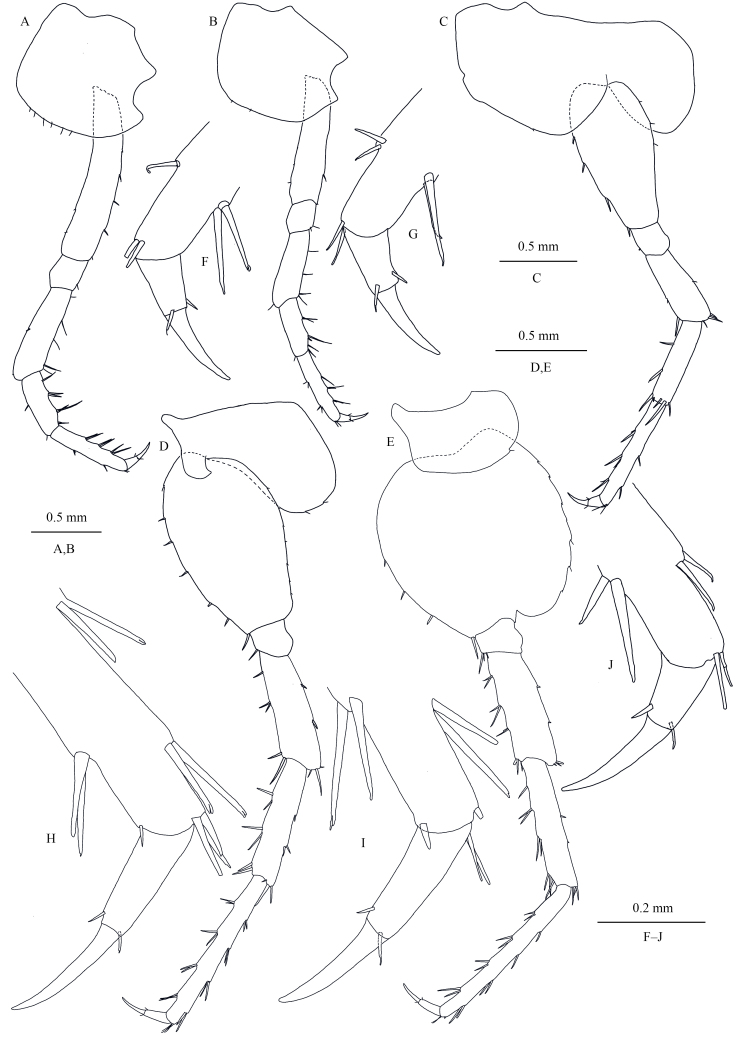
*Kachinorchestia putao* sp. nov. holotype male **A** pereopod III; **B** pereopod IV; **C** pereopod V; **D** pereopod VI; **E** pereopod VII; **F** dactylus of pereopod III; **G** dactylus of pereopod IV; **H** dactylus of pereopod V; **I** dactylus of pereopod VI; **J** dactylus of pereopod VII.

Pereopods III–VII (Fig. 5A–E), simplidactylate. Pereopod III (Fig. 5A and F): coxal plate with a posterior cusp, ventral margin with short spines; basis longest, with spines on anterior and posterior margins; merus, carpus and propodus in length ratio 1.0: 0.7: 0.9; carpus and propodus with spines on posterior margins; dactylus with two spines at hinge of unguis. Pereopod IV (Fig. 5B and G): shorter than pereopod III; coxal plate with a shorter posterior cusp, ventral margin with setae; merus, carpus and propodus in length ratio 1.0: 0.7: 1.1, dactylus with two spines at hinge of unguis. Pereopod V (Fig. 5C and H): coxal plate bilobed, anterior lobe larger than posterior lobe, bearing one seta on anterior lobe; basis suboval, with spines on both margins; merus, carpus and propodus in length ratio 1.0: 1.2: 1.7, with spines on both margins; dactylus with two spines at hinge of unguis. Pereopod VI (Fig. 5D, I): coxal plate bilobed, anterior lobe much smaller than posterior lobe, bearing two setae on posterior lobe; basis suboval, with seven spines on anterior margin and many setae on posterior margin; merus, carpus and propodus in length ratio 1.0: 1.1: 1.6, with spines on both margins; propodus and dactylus slender, dactylus with two spines at hinge of unguis. Pereopod VII (Fig. 5E and J): coxal plate unilobate, shallow; basis oval, with four spines on anterior margin and setae on posterior margin; merus, carpus and propodus in length ratio 1.0: 1.2: 1.6, with spines on both margins; propodus and dactylus slender, dactylus with two spines at hinge of unguis.

**Figure 6.**
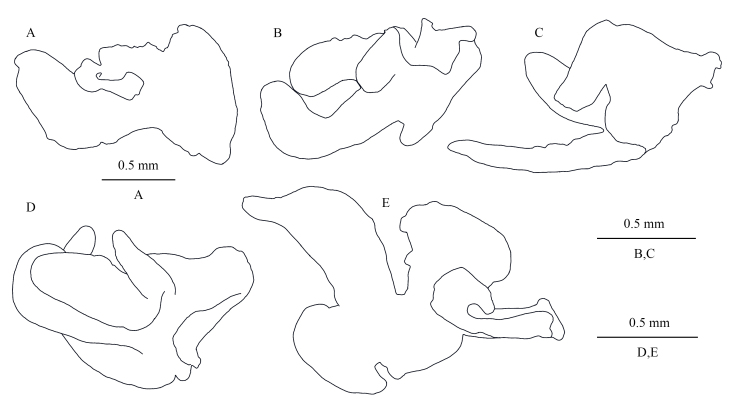
*Kachinorchestia putao* sp. nov. holotype male **A** gill of gnathopod II; **B** gill of pereopod III; **C** gill of pereopod IV; **D** gill of pereopod V; **E** gill of pereopod VI.

Coxal gills (Fig. 6A–E): present on gnathopod II and pereopods III–VI; gill of gnathopod II broad and lobed; gill of pereopods III and IV similar, lobed and convoluted; gill of pereopod V lobed and convoluted; gill of pereopod VI consisting of three lobes, middle lobe largest.

**Pleon.** Epimeral plates (Fig. 4J–L): plate I rounded posterodistally, with two fine setae on posterior margin; plates II–III acuminate posterodistally, with setae on posterior margin, respectively.

Pleopods I–III similar (Fig. 4H): peduncle with two retinacula and dense short setae on interior margin, exterior margin with plumose setae; outer ramus subequal in length to peduncle, inner ramus about 70% of outer ramus, both inner and outer rami fringed with plumose setae.

**Urosome.** Uropods I–III (Fig. 4E–G): uropod I peduncle slightly longer than ramus, inner margin with four spines and outer margin with six spines, distolateral spine distinct, longer than subdistal one; inner ramus with four marginal spines and five terminal spines; outer ramus marginally bare, with three terminal spines. Uropod II shorter than uropod I; peduncle subequal in length with rami, inner margin with three spines and outer margin with five spines; inner ramus with three marginal spines and five terminal spines; outer ramus weak, shorter than inner ramus, with one spine on interior side and some small teeth distally. Uropod III peduncle expanded, with a row of setae on ventral margin and one strong posterodistal spine; ramus narrower and shorter than peduncle, with one terminal spine.

Telson (Fig. 4I): uncleft, with one terminal spine each side.

**Description of paratype female** (IZCAS-I-A2096-2), 9.0 mm (Fig. 2).

**Figure 7.**
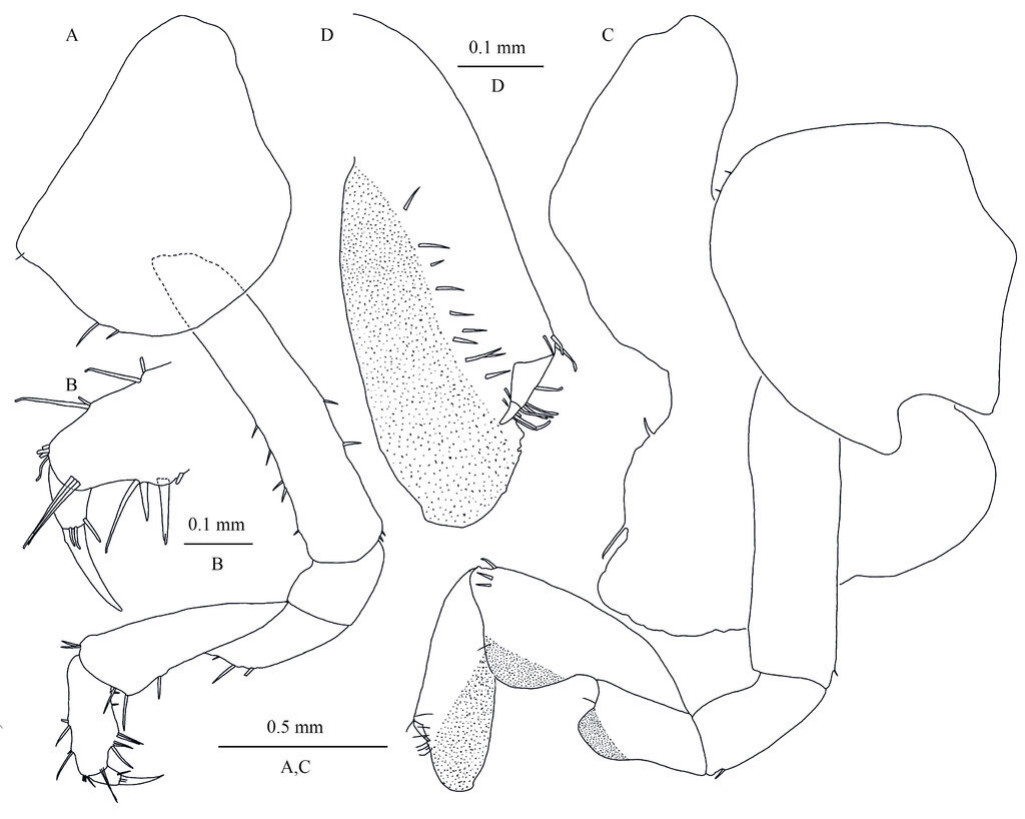
*Kachinorchestia putao* sp. nov. paratype female **A** gnathopod I; **B** propodus of gnathopod I; **C** gnathopod II; **D** propodus of gnathopod II.

**Pereon.** Gnathopod I (Fig. 7A and B): coxal plate ventral margin rounded, with three small spines; basis slender and long, with short setae on anterior and posterior margins; merus, carpus and propodus in length ratio 1.0: 2.2: 1.2; merus without protuberance humps, bearing three spines on posterior margin; carpus and propodus with spines on anterior and posterior margins; dactylus with one spine on posterior margin and two spines at hinge of unguis. Gnathopod II (Fig. 7C and D): coxal plate with cuspidate posterior margin, ventral margin spinose; basis slender, posterior margin with one spine; merus with protuberance posteriorly and one spine; carpus and propodus expanded, with protuberance posteriorly, propodus with setae on surface and palm margin; dactylus about half of the propodus palm.

**Figure 8.**
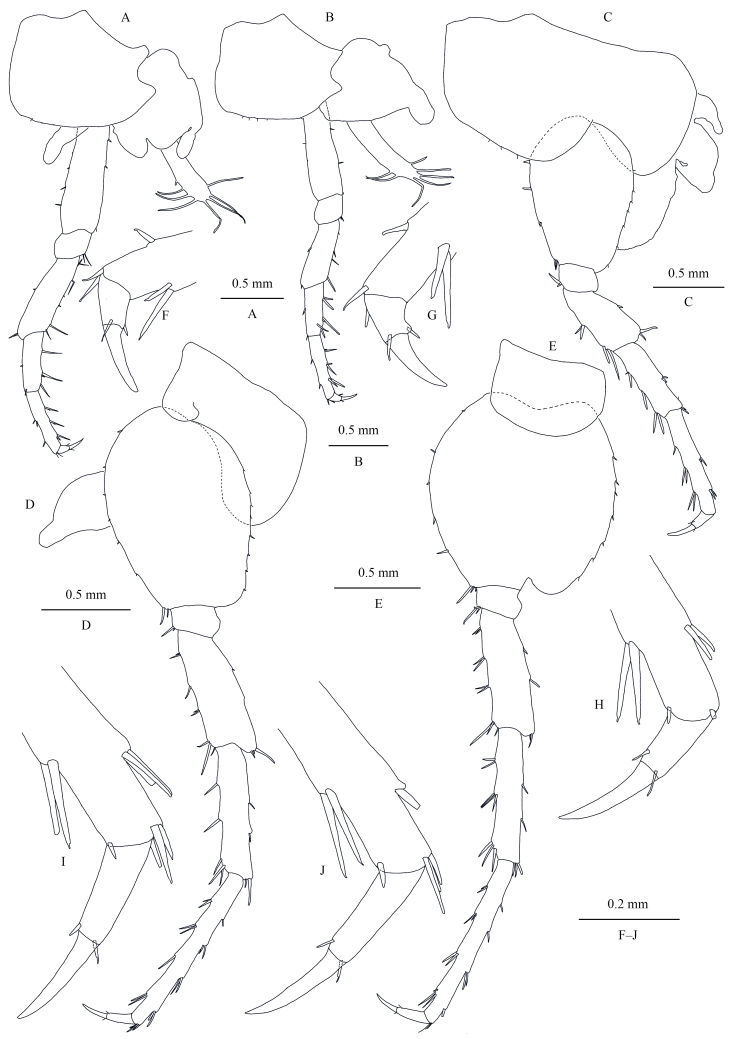
*Kachinorchestia putao* sp. nov. paratype female **A** pereopod III; **B** pereopod IV; **C** pereopod V; **D** pereopod VI; **E** pereopod VII; **F** dactylus of pereopod III; **G** dactylus of pereopod IV; **H** dactylus of pereopod V; **I** dactylus of pereopod VI; **J** dactylus of pereopod VII.

Pereopods III–VII (Fig. 8A–J): similar to those of male.

Oostegites (Fig. 8A and B): present on gnathopod II and pereopods III–V.

**Remarks**:

The new genus *Kachinorchestia* gen. nov. belongs to the Arcitalitridae family. It is most similar to the genus *Myanmarorchestia* in coxal gills convoluted, left lacinia 4-dentate, well-developed pleopods and bare outer ramus in uropod I. It can be distinguished from *Myanmarorchestia* by the following characters (*Myanmarorchestia* in parentheses): gnathopod I sexually dimorphic, propodus of male distally expanded and subchelate (not sexually dimorphic, simple); male gnathopod II propodus enlarged, oval and subchelate, with hook posteriorly and spines (transitional form, weakly chelate, with tumescence); telson uncleft (apically notched). It can be distinguished from *Solitroides* by the following characters (*Solitroides* in parentheses): pleopods well-developed (reduced) and maxilliped palp segmented (coalescing). It can be distinguished from one landhopper genus from the Philippines (Lowry and Coleman 2012), *Curiotalitrus* Lowry & Coleman, 2012 by the following characters (*Curiotalitrus* in parentheses): gnathopod I sexually dimorphic, merus of male protuberant medioposteriorly (not sexually dimorphic, not protuberant); gnathopod II sexually dimorphic, propodus of male enlarged, oval and subchelate, with hook posteriorly and spines (not sexually dimorphic, mitten-shaped); pleopods rami with numerous articles (fused 1-articulate).

*Kachinorchestia putao* sp. nov. is closely related to *M. peterjaegeri*. The similarities between the two species include gnathopods I–II sexually dimorphic, the shape of pereopods III–VII and telson with one spine on each lobe. The new species can be distinguished from *M. peterjaegeri* by the following characters (*M. peterjaegeri* in parentheses): (1) male gnathopod I merus and carpus protuberant medioposteriorly, propodus distally expanded (merus, carpus and propodus simple); (2) male gnathopod II propodus enlarged and oval, with hook posteriorly (anteroventrally produced, with tumescence); (3) coxal gills without marginal filamentous projections (with marginal filamentous projections); (4) uropod III ramus with one spine apically (with two spines); and (5) telson apically smooth with no notch (apically notched). *Kachinorchestia putao* sp. nov. can be distinguished from *M. seabri* by the following characters (*M. seabri* in parentheses): (1) male gnathopod I merus and carpus protuberant medioposteriorly, propodus distally expanded (merus, carpus and propodus simple); (2) male gnathopod II propodus enlarged and oval (with tumescence, subtriangular); (3) coxal gills without ridged margins (with ridged margins); (4) uropod III ramus with one spine apically (with two spines); and (5) telson apically without notched (apically notched). *Kachinorchestia putao* sp. nov. can be distinguished from *M. victoria* by the following characters (*M. victoria* in parentheses): (1) male gnathopod I merus and carpus protuberant medioposteriorly, propodus distally expanded (merus, carpus and propodus simple); (2) male gnathopod II propodus enlarged and oval, with hook posteriorly (with tumescence, subtriangular); (3) coxal gills without ridged margins (with ridged margins); (4) uropod III ramus with one spine apically (with two spines); and (5) telson apically smooth with no notch (apically notched). *Kachinorchestia putao* sp. nov. can be distinguished from *M. nunomurai* by the following characters (*M. nunomurai* in parentheses): (1) male gnathopod I merus and carpus protuberant medioposteriorly, propodus distally expanded, (merus, carpus and propodus simple); (2) male gnathopod II propodus enlarged and oval, with hook posteriorly (transitional form, weakly chelate); (3) gnathopod I coxal plate not produced on anterior margin (produced proximally); (4) uropod III ramus with one spine apically (with three spines); and (5) telson apically uncleft (slightly cleft apically).

**Molecular phylogeny**

**Figure 9.**
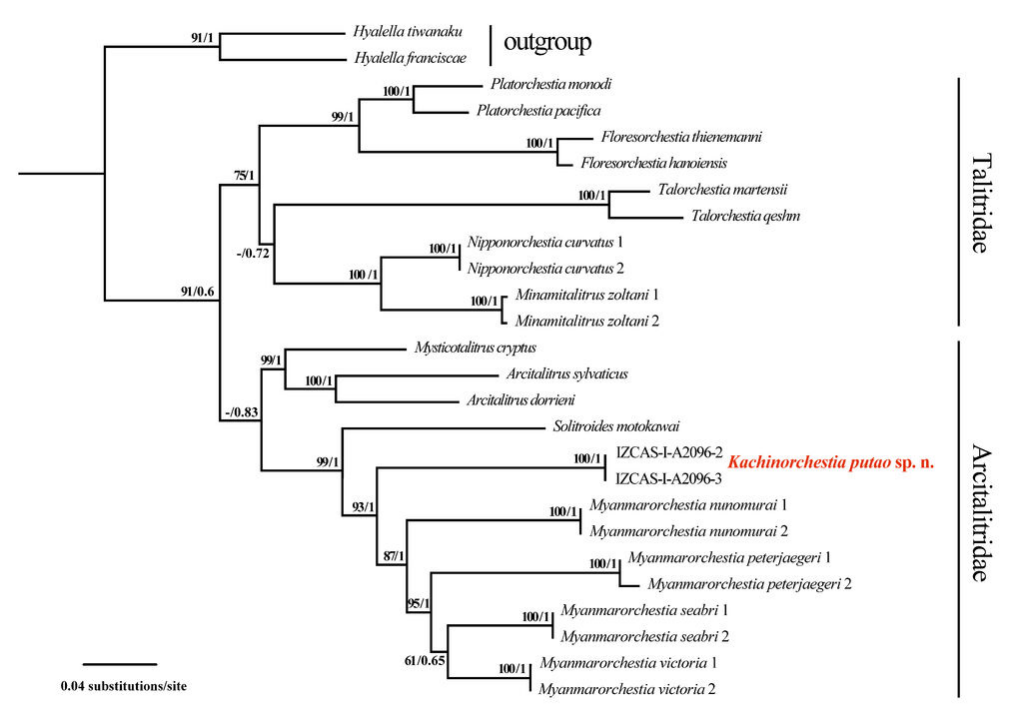
The ML tree of talitrid species generated with RAxML, based on the concatenated dataset of 28S and COI sequences. Support values are shown above branches in order for ML and BI analyses.

The final alignment contained 26 individuals with 1954 bp, including 1347 bp for 28S and 607 bp for COI. ML and BI yielded a congruent topology (Fig. 9). Our phylogeny supports the monophyly of the family Arcitalitridae, which includes two clades. One contains *Arcitalitrus* and *Mysticotalitrus*, the other clade consists of the new genus *Kachinorchestia*, *Myanmarorchestia* and *Solitroides*. This result is well supported by previous studies (Myers and Lowry 2020, Liu et al. 2023). The uncorrected pairwise distance of *Kachinorchestia putao* sp. nov. and the other four *Myanmarorchestia* species is 25.5%–27% for COI; more than 4.4% for 28S (Table 2). The divergences confirmed the distinctness of new species, in comparison with the various COI distances used for amphipod delimitation (Lefébure et al. 2006, Hou and Li 2010). In addition, the COI distance between *Kachinorchestia putao* sp. nov. and *Solitroides motokawai* is 26.8%, similar to distances within genus *Myanmarorchestia*. While genetic distance for 28S between *Kachinorchestia putao* gen. nov. and *Solitroides motokawai* is 6.9%, larger than the distance within the genus *Myanmarorchestia*. Previous study suggests that the genus *Myanmarorchestia* originated from coastal ancestors and their diversification was driven by the collision between the Burma Plate and the northeast edge of the Indian Plate (Liu et al. 2023). The location of *Kachinorchestia putao* is the most inner point on the Burma Plate connected with India Plate and the Asia continent, which could be the earlier collision site transforming from coastal to montane habitat.

In summary, morphological examination, molecular phylogenetic analyses and distribution data support *Kachinorchestia* gen. nov. being a new genus. However, the biogeography of the genus *Kachinorchestia* and its morphological adaptation in convoluted gills needs to be further studied.

#### Diagnosis

Male gnathopod I merus and carpus protuberant medioposteriorly, propodus distally expanded and subchelate; male gnathopod II propodus enlarged, oval and subchelate, with hook posteriorly and spines; coxal gills without marginal filamentous projections and ridged margins; uropod III ramus with one spine apically; and telson apically uncleft.

#### Etymology

The species name is derived from the name of the type locality; noun in apposition.

#### Distribution

Myanmar (Kachin State, type locality, Fig. 1).

## Identification Keys

### Key to species of *Kachinorchestia* and *Myanmarorchestia*

**Table:** 

1	Male gnathopod II propodus massively enlarged, subchelate	*K. putao* sp. nov.
–	Male gnathopod II mitten-shaped (parachelate)	2
2	Uropod III peduncle with a single long posterodistal robust seta	3
–	Uropod III peduncle with two unequal length posterodistal robust setae	*M. seabri* Hou, 2017
3	Pereopod VII much shorter than pereopod VI	*M. nunomurai* Nakano & Morino, 2018
–	Pereopod VII subequal with or longer than pereopod VI	4
4	Uropod II outer ramus only a little short than inner ramus, with three or four apical robust setae and no marginal setae	*M. peterjaegeri* Hou, 2017
–	Uropod II outer ramus much shorter than inner ramus, with one marginal and one apical robust seta	*M. victoria* Hou, 2017

## Supplementary Material

XML Treatment for Kachinorchestia

XML Treatment for Kachinorchestia putao

## Figures and Tables

**Table 1.** Samples used for the phylogenetic analyses, with locality information accompanied by sequence accession numbers.

**Species**	**Locality**	**COI**	**28S**
*Kachinorchestia putao***sp. nov.** (IZCAS-I-A2096-2)	Myanmar	PV764417	PV987539
*Kachinorchestia putao***sp. nov.** (IZCAS-I-A2096-3)	Myanmar	PX315801	PX316576
*Myanmarorchestia nunomurai* 1	Myanmar	OQ512653	OQ514792
*Myanmarorchestia nunomurai* 2	Myanmar	PV760292	OQ514791
*Myanmarorchestia peterjaegeri* 1	Myanmar	OQ512620	OQ514768
*Myanmarorchestia peterjaegeri* 2	Myanmar	OQ512619	OQ514876
*Myanmarorchestia seabri* 1	Myanmar	OQ512626	OQ514773
*Myanmarorchestia seabri* 2	Myanmar	OQ512630	OQ514776
*Myanmarorchestia Victoria* 1	Myanmar	OQ512624	OQ514771
*Myanmarorchestia Victoria* 2	Myanmar	OQ512623	OQ514770
*Platorchestia monodi*	Australia	OQ512228	OQ514574
*Platorchestia pacifica*	Russia	OQ512217	OQ514566
*Floresorchestia thienemanni*	Indonesia	OQ512301	OQ514373
*Floresorchestia hanoiensis*	Singapore	OQ512304	OQ514620
*Talorchestia martensii*	Indonesia	OQ512422	OQ514419
*Talorchestia qeshm*	Iran	MG655858	MG655800
*Nipponorchestia curvatus* 1	Japan	-	LC566566
*Nipponorchestia curvatus* 2	Japan	LC566563	LC566562
*Minamitalitrus zoltani* 1	Japan	LC566559	LC566558
*Minamitalitrus zoltani* 2	Japan	LC566555	LC566554
*Mysticotalitrus cryptus*	Australia	OQ512596	OQ514482
*Arcitalitrus sylvaticus*	Australia	OQ512532	OQ514451
*Arcitalitrus dorrieni*	New Zealand	OQ512551	OQ514460
*Solitroides motokawai*	Vietnam	LC223808	LC223807
*Hyalella tiwanaku*	Xinjiang, China	OM513861	MW047142
*Hyalella franciscae*	Beijing, China	OM513891	MW047137

**Table 2.** GenBank accession numbers and uncorrected pairwise distances of the COI and 28S partial sequences between *Kachinorchestia* and *Myanmarorchestia* species.

**Species**	**28S**	**1**	**2**	**3**	**4**	**5**	**6**
	**COI**	OQ514781	OQ514791	OQ514768	OQ514776	OQ514771	LC223807
1 *K. putao***sp. nov.**	PV764417		0.055	0.044	0.048	0.051	0.069
2 *M. nunomurai*	PV760292	0.255		0.044	0.046	0.041	0.072
3 *M. peterjaegeri*	MF663279	0.268	0.231		0.013	0.030	0.078
4 *M. seabri*	MF663278	0.270	0.255	0.188		0.031	0.078
5 *M. victoria*	MF969263	0.255	0.222	0.175	0.148		0.069
6 *S. motokawai*	LC223808	0.268	0.266	0.233	0.244	0.230	
